# Glutathione deficiency down-regulates hepatic lipogenesis in rats

**DOI:** 10.1186/1476-511X-9-50

**Published:** 2010-05-19

**Authors:** Corinna Brandsch, Tobias Schmidt, Diana Behn, Kristin Weiße, Andreas S Mueller, Gabriele I Stangl

**Affiliations:** 1 Institute of Agricultural and Nutritional Sciences, Martin-Luther-University Halle-Wittenberg, Von-Danckelmann-Platz 2, D-06120 Halle (Saale), Germany

## Abstract

**Background:**

Oxidative stress is supposed to increase lipid accumulation by stimulation of hepatic lipogenesis at transcriptional level. This study was performed to investigate the role of glutathione in the regulation of this process. For that purpose, male rats were treated with buthionine sulfoximine (BSO), a specific inhibitor of γ-glutamylcysteine synthetase, for 7 days and compared with untreated control rats.

**Results:**

BSO treatment caused a significant reduction of total glutathione in liver (-70%), which was attributable to diminished levels of reduced glutathione (GSH, -71%). Glutathione-deficient rats had lower triglyceride concentrations in their livers than the control rats (-23%), whereas the circulating triglycerides and the cholesterol concentrations in plasma and liver were not different between the two groups of rats. Livers of glutathione-deficient rats had lower mRNA abundance of sterol regulatory element-binding protein (SREBP)-1c (-47%), Spot (S)14 (-29%) and diacylglycerol acyltransferase 2 (DGAT-2, -27%) and a lower enzyme activity of fatty acid synthase (FAS, -26%) than livers of the control rats. Glutathione-deficient rats had also a lower hepatic activity of the redox-sensitive protein-tyrosine phosphatase (PTP)1B, and a higher concentration of irreversible oxidized PTP1B than control rats. No differences were observed in protein expression of total PTP1B and the mature mRNA encoding active XBP1s, a key regulator of unfolded protein and ER stress response.

**Conclusion:**

This study shows that glutathione deficiency lowers hepatic triglyceride concentrations via influencing lipogenesis. The reduced activity of PTP1B and the higher concentration of irreversible oxidized PTP1B could be, at least in part, responsible for this effect.

## Background

Non-alcoholic fatty liver disease (NAFLD) affects approximately 20-30% of the population in developed countries and is a common finding in patients with metabolic syndrome [1,2]. Besides enhanced lipolysis and decreased β-oxidation, NAFLD is supposed to be caused also by stimulated lipogenesis [3]. Sterol regulatory element-binding protein (SREBP)-1c is a key transcription factor in controlling the mRNA expression of genes which determine lipogenesis [4]. Since oxidative stress is generally participating in the development and progression of diabetes and its complications [5-7], it was assumed that triglyceride accumulation in the liver might be, at least in part, induced by oxidative stress [8]. Actually, recent findings showed that human hepatoma HepG2 cells which were treated with H_2_O_2 _accumulated triglycerides through up-regulation of genes encoding SREBP-1c and other genes involved in fatty acid metabolism [8], and experiments from our research group revealed a higher mRNA expression of fatty acid synthase (FAS), glucose-6-phosphate dehydrogenase (G6PDH) and stearoyl-CoA desaturase (SCD)-1 in HepG2 cells treated with pro-oxidant CuSO_4 _compared to untreated cells [9]. Data from both studies indicate oxidative stress as a stimulator of lipid synthesis in liver. However, in contrast to these findings, low levels of glutathione induced by administration of buthionine sulfoximine (BSO), a specific inhibitor of γ-glutamylcysteine synthetase [10], have been shown to attenuate ethanol-induced steatosis as well as hepatic triglyceride concentrations in untreated rats [11]. Glutathione is the most abundant thiol antioxidant in mammalian cells that is directly involved in defense of reactive oxygen species and that functions as a cofactor of antioxidant enzymes such as the glutathione peroxidase (GPx) [12]. Although pro-oxidants and many pathological conditions such as inflammatory liver diseases, diabetes and hyperglycemia are accompanied by reduced intracellular levels of glutathione [13-15], the effect of inhibited glutathione synthesis as a model for endogenously produced oxidative stress on lipogenesis is not yet well understood.

This study investigated the effect of glutathione depletion on lipid concentrations in plasma and liver, on expression of genes and activities of enzymes involved in lipid synthesis. Glutathione levels were reduced by administration of BSO. Treatment of animals with BSO has the advantage to lower tissue glutathione levels without any overt toxicity [16] or any effect on the hepatic microsomal and cytosolic enzymes [16,17]. Lipid synthesis was investigated at the transcriptional level by the analysis of the mRNA expression of SREBP-1c, the key transcription factor involved in the stimulation of lipogenesis in the liver [18,19], and of related enzymes involved in lipid synthesis and at the activity level by analysis of lipogenic enzymes FAS and G6PDH.

We assume that protein-tyrosine phosphatase (PTP)1B could play a crucial role in the effect of glutathione depletion on lipid metabolism, because PTP1B is a redox-sensitive protein and it has been identified as a potent inductor of SREBP-1 gene expression and as a novel regulator of lipogenesis [20]. In active PTP1B, the catalytic cysteine Cys215 is reduced [21,22], whereas oxidation of Cys215 in the presence of mild oxidative conditions causes the reversible formation of a cyclic sulfenamide species and leads to the inhibition of enzyme activity [23,24]. Reducing agents such as glutathione have been shown to reduce the active site sulfenamide and fully restore the PTP1B activity [23,24]. On the other hand, in addition to sulfenamide bond formation, glutathionylation of the catalytic cysteine in PTP1B might also protect the enzyme against overoxidation [25]. Based on those findings, we addressed the question whether glutathione depletion had modulated lipid metabolism via altered PTP1B activity. We further investigated the activity of the transcription factor X-box binding protein (XPB)1 that has been identified as a key regulator of the mammalian unfolded protein response as well as a stimulator of hepatic lipogenesis [26] and the inositol-requiring enzyme (IRE)-1 that transforms the XBP1 mRNA in its active form (XBP1s) by splicing.

## Results

### Body and liver weights, glutathione status and activities of antioxidative enzymes in liver

Rats treated with BSO had lower body weights than the control rats, although each rat received an equal amount of food (Table 1, P < 0.05). Liver weight was not different between the two groups of rats. In liver, glutathione was mainly present in the reduced form (GSH), whereas concentrations of the oxidized form (GSSG) were very low. The livers of rats treated with BSO had 70% lower concentrations of total glutathione than the livers of the control rats (Table 1, P < 0.05). The reduction in hepatic glutathione level was mainly due to a significant reduction of GSH (P < 0.05), whereas GSSG was not altered by the treatment. The hepatic GSH:GSSG-ratio was reduced by 64% in the BSO-treated rats compared to the control rats (P < 0.05). The activity of glutathione reductase in liver was higher in rats treated with BSO compared to the controls (Table 1, P < 0.05), whereas the enzyme activities of GPx, superoxide dismutase (SOD) and catalase did not differ between the two groups of rats.

**Table 1:** Body weight, total, reduced and oxidized glutathione and enzyme activities in control and BSO rats.

	Control group	BSO group
Final body weight (g)	225 ± 13	204 ± 12*
Liver weight (g)	8.87 ± 0.62	9.17 ± 0.89
Liver		
Total glutathione (μmol/g)	5.39 ± 0.69	1.61 ± 0.63*
GSH^a ^(μmol/g)	5.28 ± 0.70	1.53 ± 0.61*
GSSG (μmol/g)	0.05 ± 0.02	0.04 ± 0.02
GSH:GSSG-ratio	108 ± 46	42 ± 16*
GPx (U/mg protein)	1.69 ± 0.67	1.48 ± 0.45
Glutathione reductase (mU/mg protein)	64.9 ± 10.8	95.2 ± 14.8*
SOD (U/mg protein)	48.6 ± 8.5	56.2 ± 10.4
Catalase (U/mg protein)	1421 ± 222	1307 ± 373

Values are means ± SD, n = 10. * Different from control rats at P < 0.05, determined by Student's *t*-test. ^a ^GPx, glutathione peroxidase, GSH, reduced glutathione; GSSG, oxidized glutathione; SOD, superoxide dismutase.

### Lipid concentrations in plasma, lipoproteins, and liver

Concentrations of triglycerides in plasma and very low density lipoproteins (VLDL) were not different between the two groups of rats (Table 2). In liver, the concentration of triglycerides was lower in rats fed BSO than in control rats (Table 2, P < 0.05), whereas the concentrations of cholesterol in plasma, low density lipoproteins (LDL), high density lipoproteins (HDL) and liver were not different between the two groups of rats (Table 2).

**Table 2:** Concentrations of triglycerides and cholesterol in plasma, lipoproteins and liver of control and BSO rats.

	Control group	BSO group
Triglycerides		
Plasma (mmol/l)	1.66 ± 0.47	2.02 ± 0.47
VLDL (mmol/l)	0.79 ± 0.23	0.91 ± 0.38
Liver (μmol/g)	26.1 ± 2.8	20.2 ± 5.2*
Cholesterol		
Plasma (mmol/l)	2.24 ± 0.40	2.09 ± 0.42
LDL (mmol/l)	0.31 ± 0.06	0.34 ± 0.12
HDL (mmol/l)	1.22 ± 0.17	1.18 ± 0.30
Liver (μmol/g)	7.82 ± 0.88	6.77 ± 1.40

Values are means ± SD, n = 10. * Different from control rats at P < 0.05, determined by Student's *t*-test.

### mRNA concentrations and activities of enzymes involved in hepatic lipogenesis

**Figure 1:**
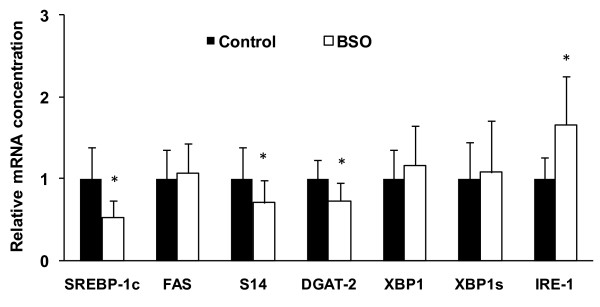
**Hepatic mRNA concentrations of genes involved in regulation of lipogenesis in control and BSO rats**. Glyceraldehyde-3-phosphate dehydrogenase was used for normalization, values of BSO rats were related to values of control rats (= 1.00). Values are means ± SD, n = 10; *Different from control rats at P < 0.05, determined by Student's *t*-test. DGAT, diacyl glycerol acyl transferase; FAS, fatty acid synthase; IRE, inositol requiring enzyme; SREBP, sterol regulatory element-binding protein; S14, Spot 14; XBP1, X-box binding protein 1; XBP1s, splicing variant of XBP1.

**Figure 2:**
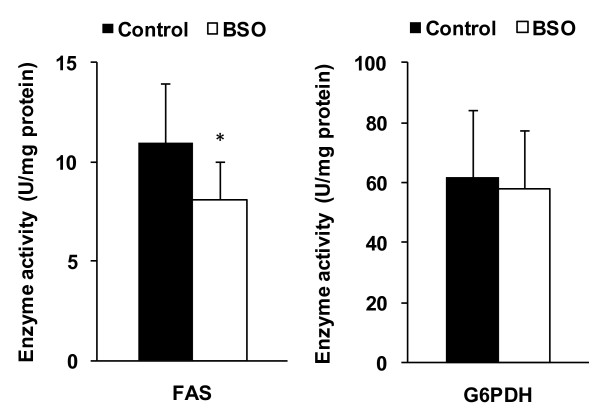
**Activities of lipogenic enzymes in liver of control and BSO rats**. Values are means ± SD, n = 10. *Different from control rats at P < 0.05, determined by Student's *t*-test. FAS, fatty acid synthase; G6PDH, glucose-6-phosphate dehydrogenase.

The mRNA abundance of SREBP-1c, DGAT2 and S14 in livers of BSO treated rats was significantly lower than in livers of the control rats, whereas the transcriptional level of FAS did not differ between the two groups (Figure 1, P < 0.05). Despite higher mRNA concentrations of IRE-1 in livers of BSO treated rats compared to the controls (P < 0.05), the mRNA concentrations of XBP1 and XBP1s were not different between the two groups (Figure 1). Analysis of hepatic FAS activity reveals that the rats treated with BSO had lower activities than the control rats (P < 0.05, Figure 2). The activity of G6PDH was not different between the two groups of rats (Figure 2).

### Activity and protein concentration of PTP1B in liver

**Figure 3:**
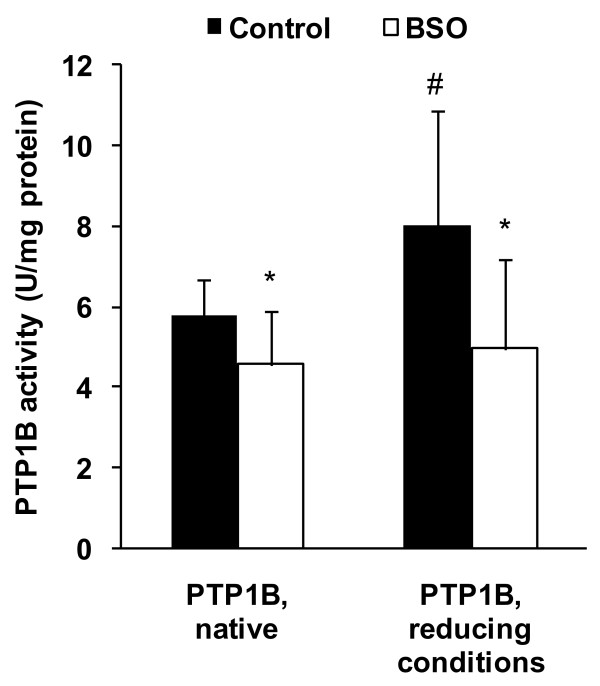
**Activity of PTP1B in liver of control and BSO rats**. Activity was measured under native and under reducing conditions (DTT). Values are means ± SD, n = 10; *Different from control rats at P < 0.05, determined by Student's *t*-test. ^#^Different from native PTP1B at P < 0.05, determined by paired *t*-test.

**Figure 4:**
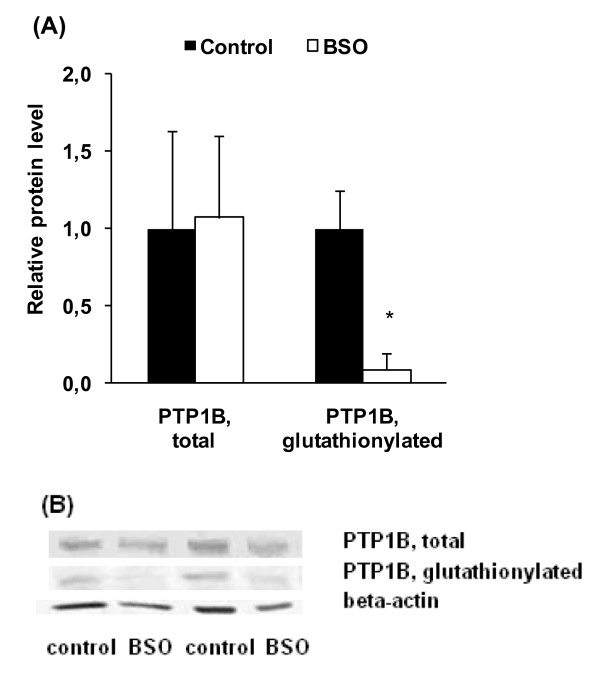
**Protein expression of total and glutathionylated PTP1B in liver of control and BSO rats**. (A) Densitometric analysis of protein abundance after Western blotting. Beta-actin was used for normalization, values of BSO rats were related to values of control rats (= 1.00). Values are means ± SD, n = 10; *Different from control rats at P < 0.05, determined by Student's *t*-test. (B) Representative bands of total and glutathionylated PTP1B and beta-actin.

The native activity (under non-reducing conditions) and the activity of PTP1B under reducing conditions (DTT) were lower in livers of rats treated with BSO than in the controls (Figure 3, P < 0.05). In the livers of control rats the activity of PTP1B under reducing conditions was higher than under native conditions, whereas those differences were not seen in the livers of rats treated with BSO (Figure 3, P < 0.05). The total protein concentration of PTP1B in the liver was not different between the two groups of rats, but the concentration of glutathionylated PTP1B was markedly lower in the BSO group than in the control group (Figure 4, P < 0.05).

## Discussion

This study investigated the effect of glutathione deficiency on lipid metabolism. Cellular GSH may be decreased by administering compounds that react with GSH to form conjugates or that oxidize GSH to GSSG. However, these approaches are limited by lack of specificity of the reagents available. Moreover, the effects obtained are transient, associated with major perturbations of metabolism, or both. In this study we used BSO, a relatively nontoxic inhibitor of γ-glutamylcysteine synthetase, to cause a decrease in glutathione level. The effects observed after treatment with BSO are produced by the reactive species that are formed in normal metabolism. Thus, the BSO model of oxidative stress differs significantly from those in which oxidative stress is produced by radiation or by giving compounds that produce oxidation. As expected, treatment with BSO for 7 days caused a significant depletion of total glutathione in the liver of the rats. The diminished levels of total glutathione were mainly attributable to a reduction of GSH, whereby, in both groups of animals the intracellular level of GSSG was very small. The GSH:GSSG-ratio, which is used as an indicator of the cellular redox state [13], was markedly reduced in response to BSO treatment. Since glutathione functions as a cofactor of GPx [12], it appears plausible that the livers of BSO-treated rats showed a compensatory increase of glutathione reductase activity.

Glutathione has multiple functions ranging from antioxidant defense to modulation of immune function and many conditions are related to low glutathione levels [12,27]. Glutathione concentration has been found to be decreased in chemically induced diabetic animals [e.g. 28,29], and transcriptional level of γ-glutamylcysteine synthetase has been found to be lowered in response to insulin deficiency [30] and hyperglycemia [31]. BSO-induced glutathione deficiency in this study actually lowered triglyceride concentrations in liver by 23%. These data confirm recent results of Donohue et al. [11] who found a BSO-induced reduction of triglyceride concentration in livers of healthy rats or rats with ethanol-induced steatosis. The reduced mRNA concentrations of SREBP-1c, S14, DGAT2 and diminished activity of FAS which we observed in the livers of BSO-treated rats indicate that the lower hepatic triglyceride concentration was caused by a diminished lipogenesis. Despite the lower liver lipid concentrations, the concentrations of circulating triglycerides were not lower than those of the control rats. Although there have been published a few studies that used a genetic mouse model of chronic glutathione depletion [e.g. 32-34], none of these studies had characterized the lipid metabolism in those animals. A histological survey revealed no evidence for abnormalities in livers of such knockout mice [32], albeit hepatic triglycerides were not measured in this context.

In contrast to our results, there are a couple of other studies which observed a stimulation of lipogenesis in response to oxidative stress or ER stress. In particular, experiments with human hepatoma cells (HepG2) showed that oxidative stress induced by H_2_O_2 _[8] or copper [9] leads to triglyceride accumulation in these cells by up-regulation of lipogenic transcription factors and enzymes involved in fatty acid synthesis. In addition, Lin et al. [35] found that oxidative ER stress which was induced by administration of a high saturated fat diet stimulates lipogenesis in liver of mice, whereas antioxidative cysteine-containing compounds such as n-acetyl cysteine were capable of down-regulating lipogenesis. As previously mentioned, we assume that the BSO model which is a model for endogenously produced oxidative stress in normal metabolism differs significantly from those in which oxidative stress is produced by administered compounds that produce exogenous oxidation. However, it would be an interesting scientific issue, how pro-oxidants would influence lipid metabolism under conditions of glutathione deficiency. Based on our findings, we assume that the reduced activity of PTP1B could by an explanation for the lower triglyceride concentrations in livers of BSO-treated rats. As stated before, PTP1B is a redox-sensitive key enzyme which is capable of stimulating the fatty acid synthesis via activation of SREBP-1 [20]. PTP1B is regulated via peroxide-mediated oxidation of the active-site thiol residue to a sulfonic acid [36], followed by the reaction with glutathione to a mixed disulfide, termed glutathionylation [37,38]. Glutathionylation of cysteine in the catalytic centre (Cys 215) of PTP1B leads to a reversible inactivation of the enzyme [25], but glutathionylation of the catalytic cysteine in PTP1B might also protect the enzyme against irreversible overoxidation [25]. The addition of DTT is normally used to ascertain the amount of reversible oxidized PTP1B [39]. However, our analysis revealed reversible oxidized PTP1B only in livers of untreated rats, whereas treatment with BSO increased the amounts of irreversibly oxidized PTP1B. We also observed considerably lower concentration of glutathionylated PTP1B in the BSO-treated rats, which indicates a lower protection of PTP1B against irreversible overoxidation.

On the other hand, we assume that XBP1 has not contributed to the observed alterations of lipid metabolism in the BSO-treated rats. Despite the observed up-regulation of the kinase IRE1 in livers of rats treated with BSO, the mRNA expression of XBP1 and XBP1s was not altered in these livers. IRE-1 is an ER-localizing proximal sensor of ER stress, and normally induces an unconventional splicing of XBP1 mRNA to generate a mature mRNA encoding the active transcription factor XBP1s [40,41]. XBP1s has been identified as a key regulator of ER stress response [42] and a transcriptional activator of lipogenesis [26]. The failing increase of mature XBP1s mRNA expression probably contributed, that we did not observe any triglyceride accumulation in livers of rats treated with BSO compared to the controls.

## Conclusions

This study confirmed that short-term reduction of available glutathione under conditions of a normal metabolism lowers the concentration of triglycerides in liver probably by reduction of lipid synthesis. We assume that the reduced hepatic activity of the redox-sensitive PTP1B, the lower concentration of glutathionylated PTP1B and the higher concentration of irreversible oxidized PTP1B in the glutathione-depleted rats could explain, at least in part, the reduced levels of triglycerides in the liver of these animals.

## Materials and methods

### Animals and treatment

Twenty male 5-wk old Sprague-Dawley rats (Charles River, Sulzfeld, Germany) were randomly assigned to 2 groups of 10 each (initial body weight: 169 ± 12 g). All rats were kept individually in Macrolon cages in a room with controlled temperature (22 ± 2°C), relative humidity (50-60%), and light (12:12-h light:dark cycle). The rats were fed 16 g per day of a semi-synthetic diet consisting of (g/kg) casein (200), starch (388), sucrose (200), lard (100), cellulose (50), vitamin and mineral mixture (60), and DL-methionine (2). Vitamins and minerals were supplemented according to recommendations of the American Institute of Nutrition for rat diets [43]. Nipple drinkers allowed free access to water. All experimental procedures described followed established guidelines for the care and use of laboratory animals and were approved by the council of Saxony-Anhalt, Germany (42502/3-468 MLU).

One rat group received L-buthionine-(*S,R*)-sulfoximine (Sigma-Aldrich, Steinheim, Germany) *via *drinking water at a concentration of 20 mM yielding an average dose level of 1.77 mmol/kg body mass per day for a total of 7 days. The rats of the control group were offered drinking water without BSO. After 7 days, the rats were killed by decapitation under light anesthesia with diethyl ether. Each rat received 7 g of the diet 4 h before decapitation as short-term food-deprivation significantly down-regulates the transcriptional levels of genes encoding enzymes of the lipid metabolism [44,45] which were to be measured in this study. Whole blood was collected into heparinized polyethylene tubes and plasma was separated by centrifugation at 1,500 × *g *for 10 min at 4°C. Livers were excised, washed with ice cold NaCl solution and weighed. Aliquots of liver for RNA isolation were immediately snap frozen in liquid nitrogen and stored at -80°C. Plasma was stored at -20°C. Aliquots of liver were homogenized in ice-cold 0.1 M phosphate buffer (pH 7.4) (1:5, w/v) using a Potter-Elvehjem homogenizer and then centrifuged at 700 × g for 10 min at 4°C. The supernatant was used as crude homogenate. For analysis of enzyme activities in cytosol fractions, aliquots of the crude homogenate were centrifuged at 100,000 × g for 1 h at 4°C.

### Analysis of lipids in plasma and liver

Chylomicrons, VLDL, LDL and HDL were separated by step-wise ultracentrifugation (Mikro-Ultracentrifuge, Sorvall Products, Bad Homburg, Germany) by appropriate density cuts (chylomicrons, < 0.95 kg/l; VLDL, 0.95 <*p *< 1.006 kg/l; LDL, 1.006 <*p *< 1.040 kg/l; HDL, *p *> 1.063 kg/l). Plasma densities were adjusted by NaCl and KBr. Chylomicrons were isolated by centrifugation at 100,000 × g for 10 min at 4°C and were removed. The other fractions were separated at 900,000 × g for 1.5 h each at 4°C. Lipids from liver were extracted with a mixture of n-hexane and isopropanol (3:2, v/v) [46]. After evaporation of the solvent, the lipids were dissolved with Triton-X [47]. Concentrations of cholesterol and triglycerides in plasma, lipoproteins and lipid extracts from the liver were determined using enzymatic reagent kits (DiaSys Diagnostic Systems, Holzheim, Germany, Cat.-No. 1.1300 99 90 314 and 1.5760 99 90 314).

### Analysis of glutathione in liver

Hepatic concentrations of total glutathione, its reduced and oxidized form were measured by use of a spectrophotometric method [48]. In brief, fresh homogenates were diluted in phosphate buffer, mixed with trichloroacetic acid (10%), and centrifuged at 10,000 × g for 15 min at 4°C. The protein-free supernatant was used for analysis. Calibration was performed using standard curves. For measurement of GSSG, the tissue samples were mixed with 2-vinylpyridin and incubated for 60 min prior to analysis.

### Analysis of enzyme activities

Enzyme activities in liver were assayed by spectrophotometric methods. Catalase activity was determined at 25°C using hydrogen peroxide as substrate [49]. One unit of catalase activity is defined as the amount consuming one μmol hydrogen peroxide per min. Total SOD activity was determined according to the method of Marklund and Marklund [50] with pyrogallol as substrate. One unit of SOD activity is defined as the amount of enzyme required to inhibit the autoxidation of pyrogallol by 50%. GPx activity was determined at 25°C by use of t-butyl hydroperoxide as substrate [51]. One unit of GPx activity is defined as one μmol reduced β-nicotinamide adenine dinucleotide phosphate (NADP) oxidized per min. Glutathione reductase activity was measured according to a method of Carlberg and Mannervik [52] with one unit defined as the amount of enzyme required for the reduction of one μmol NADPH per min at 25°C. FAS activity was measured in liver cytosol by analyzing the rate of malonyl-CoA-dependent NADPH oxidation [53]. One unit of FAS activity is expressed as one nmol of NADPH oxidized per min at 25°C. G6PDH activity in liver cytosol was assayed by analyzing the rate of NADP reduction under conditions of inhibition of 6-phosphogluconate dehydrogenase by maleimide [54]. One unit of G6PDH activity is defined as one nmol of NADP reduced per min at 25°C. The PTP1B activity in liver homogenate was measured under reducing and non-reducing conditions [55] with modifications [39,56] using para-nitrophenyl phosphate (pNPP, extinction coefficient 17.8 l × M^-1 ^× cm^-1^) as substrate. Native PTP activity was measured under non-reducing conditions. To determine the total PTP activity including the reversible inactive form, the measurement was repeated under reducing conditions by adding 2.5 mmol/l of DTT to the assay buffer. All enzyme activities were normalized to 1 mg of protein. The protein contents of the samples were determined according to the method of Bradford et al. [57].

### Western blot analysis of PTP1B

Total and glutathionylated PTP1B protein abundance in liver tissue was measured by Western blot analysis according to a recently described method [39] with modifications. For analysis crude homogenates were diluted 1:10 (w/v) in RIPA lysis buffer containing 50 mM Tris-base (pH 7.4), 150 mM NaCl, 1 mM EDTA, 1% (v/v) protease inhibitor cocktail (Sigma), 1% (w/v) sodium desoxycholeate, 1% (w/v) Triton X-100, 0.1% (w/v) SDS. The homogenate was centrifuged at 10,000 × g for 30 min at 4° C, and the supernatant was removed and stored at -80° C until further processing. The protein content of the samples was determined by the bicinchoninic acid assay. 40 μg of protein per lane were separated on a 12.5% SDS-polyacrylamide gel [58] under non-reducing conditions. The separated proteins were transferred to a nitrocellulose membrane (BioTrace™ NT, Pall Corporation, Pensacola, FL, USA) by wet-blotting (90 min at 350 mA). After blocking the membranes in TBST buffer (100 mM Tris-base, pH 7.6, 150 mM NaCl, 0.05% Tween) containing 5% (w/v) non-fat dry milk overnight at 4°C, the blots were incubated with primary antibodies. For total PTP1B protein analysis the mouse anti-PTP1B antibody (BD Biosciences, Pharmingen), and for analysis of the glutathionylated PTP1B protein abundance the mouse anti-GSH antibody (Virogen, Watertown, USA) was used, both diluted 1:2,500 in TBS buffer. Incubation was carried out for 2 h at room temperature. The secondary antibody (goat anti-mouse alkaline phosphatase conjugated antibody, BioFX Lab., Owings Mills, USA) was employed at a dilution of 1:2,500 in TBS buffer for 1 h at room temperature. Antibody binding was visualized with a reaction buffer (100 mM Tris-base, 100 mM NaCl, 50 mM MgCl_2_) containing 1 M nitro-blue tetrazolium and 1 M 5-bromo-4-chloro-3-indoylphosphate. Optical densities of the bands were evaluated. For normalization a mouse anti-beta-actin antibody (Abcam, Cambridge, UK) was used (1:5,000).

### RNA isolation and real-time detection RT-PCR

The RNA was isolated from liver samples using TRIZOL™ reagent (Invitrogen, Karlsruhe, Germany) according to the manufacturer's protocol. Total RNA concentration and purity were estimated from the optical density at 260 and 280 nm, respectively. Synthesis of cDNA and determination of mRNA abundance of SREBP-1c, FAS (EC 2.3.1.85), DGAT-2 (EC 2.3.1.20), S14, PTB1B (EC 3.1.3.48), XBP1 in native and spliced form, IRE-1 and glyceraldehyd-3-phosphat dehydrogenase (GAPDH) were performed by real-time detection PCR as recently described in detail [9]. The primer sequence was for DGAT-2 For-5'-CAGCCCTTAGTGACTCAG-3', Rev-5'-GTGTACAGGAGGCCAGG-3', for FAS For-5'-AGGTGCTAGAGGCCCTGCTA-3', Rev-5'-GTGCACAGACACCTTCCCAT-3', for GAPDH For-5'-GCATGGCCTTCCGTGTTCC-3', Rev-5'-GGGTGGTCCAGGGTTTCTTACTC-3', for IRE-1 For-5'-ACCCACACGGAGACCTTACC-3', Rev-5'-ACTGGTGCCAGCCTTGAGAG-3', for SREBP-1c For-5'-GGAGCCATGGATTGCACATT-3', Rev-5'-AGGAAGGCTTCCAGAGAGGA-3', for S14 For-5'-CCAGCCTCCATCACATCCTTA-3', Rev-5'-CCCCTGGCCGCTTGCTATTAC-3', for XBP1 For-5'-ATTCTGACGCTGTTGCCTCT-3', Rev-5'-CTCTGGGGAAGGACATTTGA-3' and for XBP1s For-5'-GAGTCCGCAGCAGGTG-3', Rev-5'-GTGTCAGAGTCCATGGG-3'. Relative quantification of mRNA abundance of the genes was performed using the ΔΔCt-method with GAPDH as reference gene [59]. Ct-values of the target genes and the reference gene were obtained using RotorGene Software 5.0 (Corbett Research, Mortlake, Australia). Relative mRNA abundance of the genes investigated is expressed as fold change in the BSO group compared to the control group.

### Statistical analysis

Experimental data were analyzed using the Minitab Statistical Software (Minitab, State College, PA, USA). Means of the two groups were compared by Student's *t*-test, and means of related samples by paired *t*-test. Means were considered significantly different at P < 0.05.

## Competing interests

The authors declare that they have no competing interests.

## Authors' contributions

CB participated in the design of the study and prepared the manuscript. TS carried out the study and analyzed the concentrations of glutathione and lipids, and the activities of antioxidative enzymes. DB and ASM analyzed the PTP1B activities under non-reducing and reducing conditions and performed the western blot analysis. KW performed all PCR analysis. GIS designed and supervised this experiment. All authors read and approved the final manuscript.
